# The History of the Philadelphia Fraudulent Medical Colleges

**Published:** 1880-11

**Authors:** 


### THE HISTORY OF THE PHILADELPHIA FRAUDULENT MEDICAL COLLEGES.

The history of the fraudulent medical colleges of Philadelphia is one that is humiliating to every American. It is not alone that such institutions existed, but that they existed and flourished with increasing prosperity for more than a quarter of a century. But though, on this account, some reproach falls upon all, the keenness of disgrace touches especially the city of Philadelphia. Her civil authorities had long known that this business was going on, and that ignorant men were every year being clothed with the responsibilities of the physician. Yet they did nothing in the matter. Her medical men were no less aware of the iniquity. But from her famous colleges, her historic societies, her immortal leaders in medical science, there came no efforts to clear the reputation of the city or the profession. The editor of a penny newspaper has done more than all the organized strength of the profession to destroy the diploma mills and remove the stain upon the medical pre-eminence of Philadelphia. This is not a very proud moment for the physicians of that city. She has lost her prestige as a legitimate medical centre, and has won the reputation which comes from issuing three thousand spurious diplomas.

There is every prospect now, however, that the career of these colleges is at an end. Legal processes have been instituted against the trustees of one institution; the dean of another is languishing in jail; the proprietors of a third will soon have all their powers destroyed and their charter annulled.

The manufacture of diplomas in Philadelphia began in 1853, at which time the legislature chartered the American College of Medicine. In 1858 the name was changed to the American College of Medicine in Pennsylvania and the Eclectic Medical College of Philadelphia. In the diplomas subsequently issued, sometimes one part of the above title was given and sometimes the other. Various kinds of degrees were conferred, but most of them were medical. Finally, a division of the charter and school was made. Dr. Wm. Paine took the American College and Dr. Buchanan the Eclectic Medical College. In 1865 the name of the American College was changed by the legislature to the Philadelphia University of Medicine and Surgery. Under this name the institution has flourished for fifteen years and gained a world-wide reputation. Dr. Buchanan, meanwhile, continued his own college, adding other fictitious names to it. In 1872 the State Legislature investigated these institutions. It was discovered that medical degrees were given after a few days' study, and that degrees of all kinds were sold at moderate rates. An instance was cited in which a degree of L.L.D. was conferred on a child two years old. In consequence of the revelations thus made the charters were repealed. Dr. John Buchanan, however, carried his case to the Supreme Court, and showed that the charter of his Eclectic Medical College was given before the legislature had reserved a right to repeal such instruments. The Court sustained the plea, and the college was kept on its feet. The Philadelphia University of Medicine and Surgery did not stand on the same footing with the other college. The question whether the legislature had a right to repeal its charter is a delicate one, but it seems probable, on the whole, that it had. Dr. Wm. Paine, however, did not choose to go to law about the matter. Instead of this, he took the risk, and continued operations in spite of the repeal.

At about this time the Institution was sold out to a ministerial syndicate of four. These persons ran the "University" with great success, it being under the immediate and able control of the Rev. Mr. Miller, Dean, and the Rev. Mr. Ingraham, President. Degrees of all kinds were issued, some of them very novel and ingenious. One of these confers all the privileges and immunities of a physician upon members of "The University Hospital;" another confers the privileges that belong to a student of medicine in the "University and Hospital of Philadelphia." Since 1859 over six hundred medical degrees have been issued from this "University" alone. Most of these were conferred on persons in the United States. A full list of the names is published in the *Philadelphia Record*, of June 14th. Two hundred and twenty of the persons holding these diplomas live in Pennsylvania and contribute to the elevation of the medical status of that State. New York comes next in number, and has sixty-six. Then follow various Western States, Ohio leading. Ten names are given of persons living in Germany.

While this University was thus in active work, Dr. John Buchanan, with his Eclectic Medical College and other institutions, had been no less busy. The activity of his traffic and the elasticity of his collegiate nomenclature may be best learned from the following: A reporter of the *Philadelphia Record* bought of him, in open purchase,

One diploma of M.D. from the Eclectic Medical College; two diplomas of M.D. from the American University of Philadelphia; one diploma of M.D. from the Livingstone University of America; one diploma of M.D. from the National Eclectic Medical Association; one degree of D.D. from the Livingstone University; one degree of L.L.D. from the American University, and one degree of D.C.L. from the Livingstone University.

Only one of the above institutions, the first, so far as we can learn, has even a chartered existence. The Livingstone University is located in Camden, N. J. An attempt was made to get a charter for it from the New Jersey Legislature. This attempt failed, but Dr. Buchanan ran the University just the same, using the alias of James Murray, D.D., Dean.

In addition to the Paine-Miller and the Buchanan diplomashops, a quasi-medical college, known as the Electropathic Institute, has been unearthed. By a twenty-five hours' attendance on lectures, and the payment of $50, a degree of Master of Electricity can be obtained, and was so obtained by a *Record* reporter. This degree enables the possessor to treat all diseases, upon the theory taught at the School, a theory which is so unique as to deserve explanation. It is briefly this: No medicine ever cured disease. All diseases are simply disturbances of the electrical currents of the body. Those attended with inflammation, sharp pains, or extraneous growths, are simply evidences of too much positive electricity; those attended with dull pains, degenerations, and tendency to decomposition, are evidences of too much negative electricity. To effect a cure it is only necessary to change the abnormally negative to positive, or *vice versa.* Upon these theories the Electropathic Institute did its work, and graduated its students. Strange to say, it had received the endorsement of a number of prominent and presumably intelligent Philadelphians.

The decline and fall of all these institutions began about six months ago. The Philadelphia University of Medicine and Surgery then received a thorough exposure, and its ministerial supporters were disgraced. Still the civil authorities had no evidence by which they could convict them, and had no money with which to procure evidence and institute proceedings. Meanwhile, Dr. Buchanan, with his colleges, was still selling his diplomas, and evading all attempts to capture him. A plan was, however, at last arranged by the editor of the *Record,* for entrapping the wily President. Applications under assumed names, for diplomas, were sent to him from two distant country towns. After some negotiations the diplomas were forwarded, the money for them paid, and receipts given. Further evidence of sale of diplomas was obtained from other persons, and also by personal investigation of the collegiate building. On the 9th inst. Buchanan was arrested for using the mails to defraud, thus violating the laws of the United States; he was held in $10,000 bonds. On the next day a new indictment was brought against him for violating the State law in selling diplomas. This law, enacted in 1870, makes it a misdemeanor for any institution authorized to confer degrees, to sell such degrees, the penalty being $500 fine, and an imprisonment not exceeding six months. Under this charge Buchanan was held in further bonds of $2,000. Finally, upon the evidence of some of his associate professors, who testified that he had forged their names, he was held in bonds of $1,000 for the crime of forgery.

On June 14th, legal proceedings were begun in the Court of Common Pleas, for judgment of ouster against the trustees and officers of the Philadelphia University of Medicine and Surgery. The attempt will be made to show that, for five years past, this University has exercised powers which it had no right to assume; and that this exercise of power has been of injury to the commonwealth. There are, we believe, some nice legal points in this case, but that the decision will eventually be against the University there can scarcely be a doubt. A similar process will be, or has already been, instituted against the Eclectic Medical College.

The cords of the law are now being tightly drawn around the bogus medical colleges. If there is anything left of them after the law is done, the force of an aroused public sentiment may be relied on to complete the work. Diploma-selling is stopped for the present, and Philadelphia's reputation as a medical centre may cease to have its shady side. But we cannot feel sure that the cure is yet a permanent one. The fact that diplomas can be bought, and that diploma-selling is a paying business, has been widely advertised and will become known to the not over-scrupulous, as well as to those who are honest. Permanent protection, though so urgently needed, is not yet insured. It cannot be obtained more surely than by the enactment of a wise law regulating the practice of medicine. With such a law, with civil authorities alert and medical organizations not wholly devoted to secret discussions and punctilious care in their reporting, Philadelphia may be able to purify its reputation and attain a scientific eminence which, if not supreme, will at least not be questionable. —*New York Med. Record.*

